# A challenging case of emergency redo surgery for acute type A aortic dissecting aneurysm of ascending and aortic arch with frozen elephant trunk following aortic root replacement

**DOI:** 10.1186/s13019-024-02653-7

**Published:** 2024-04-16

**Authors:** Yuh Ing Lok, Jaime Villaquiran, James Kuo

**Affiliations:** Department of Cardiothoracic Surgery, Derriford Hospital, Derriford Road, Plymouth, PL6 8DH UK

**Keywords:** Redo aortic surgery, Emergency surgery, Ascending and aortic arch dissection, Frozen elephant trunk

## Abstract

Redo ascending and aortic arch surgeries following previous cardiac or aortic surgery are associated with high risk of morbidity and mortality due to multiple factors included sternal re-entry injury, extensive aortic arch surgery, emergency aortic surgery, prolonged cardiopulmonary bypass duration, poor heart function, and patients with older age. Therefore, appropriate surgical strategies are important. We report a case of a 72-year-old gentleman with previous surgery of aortic root replacement who presented with acute Type A aortic dissecting aneurysm of ascending and aortic arch complicated with left hemothorax, which was successfully treated by emergency redo aortic surgery with frozen elephant trunk (FET) technique.

## Introduction

The incidence of redo aortic surgery is increasing due to multiple reasons, including: new aortic dissection or aneurysm, progressive aneurysm formation in non-resected dissected aortic segments during initial surgery, pseudoaneurysm formation at suture lines, prosthetic valve endocarditis, graft infection and failure of bioprosthetic valve [1, 2]. However, redo aortic arch surgery following previous cardiac or aortic surgery is associated with significant risk of operative mortality compared to primary procedure, ranging from 5 to 19% [3], which caused by numerous factors included sternal re-entry injury secondary to dense adhesion, extensive aortic arch surgery, emergency surgery, prolonged cardiopulmonary bypass duration, poor heart function, and patients with older age. Hence, appropriate surgical approaches are the keys to a successful surgery. The FET procedure provides one-stage repair of complex aortic conditions which consists of a complete aortic arch replacement with antegrade stent grafting of the proximal descending thoracic aorta. This procedure also reduces the reintervention rate by closing the distal intimal tears, promote false lumen thrombosis and aortic remodeling in the downstream aorta [4]. Here, we present a case of emergency redo aortic surgery for leaking acute Type A aortic dissecting aneurysm by utilising FET technique, the surgical steps as well as the strategies to deal with intraoperative difficulties.

## Case presentation

**Fig. 1:**
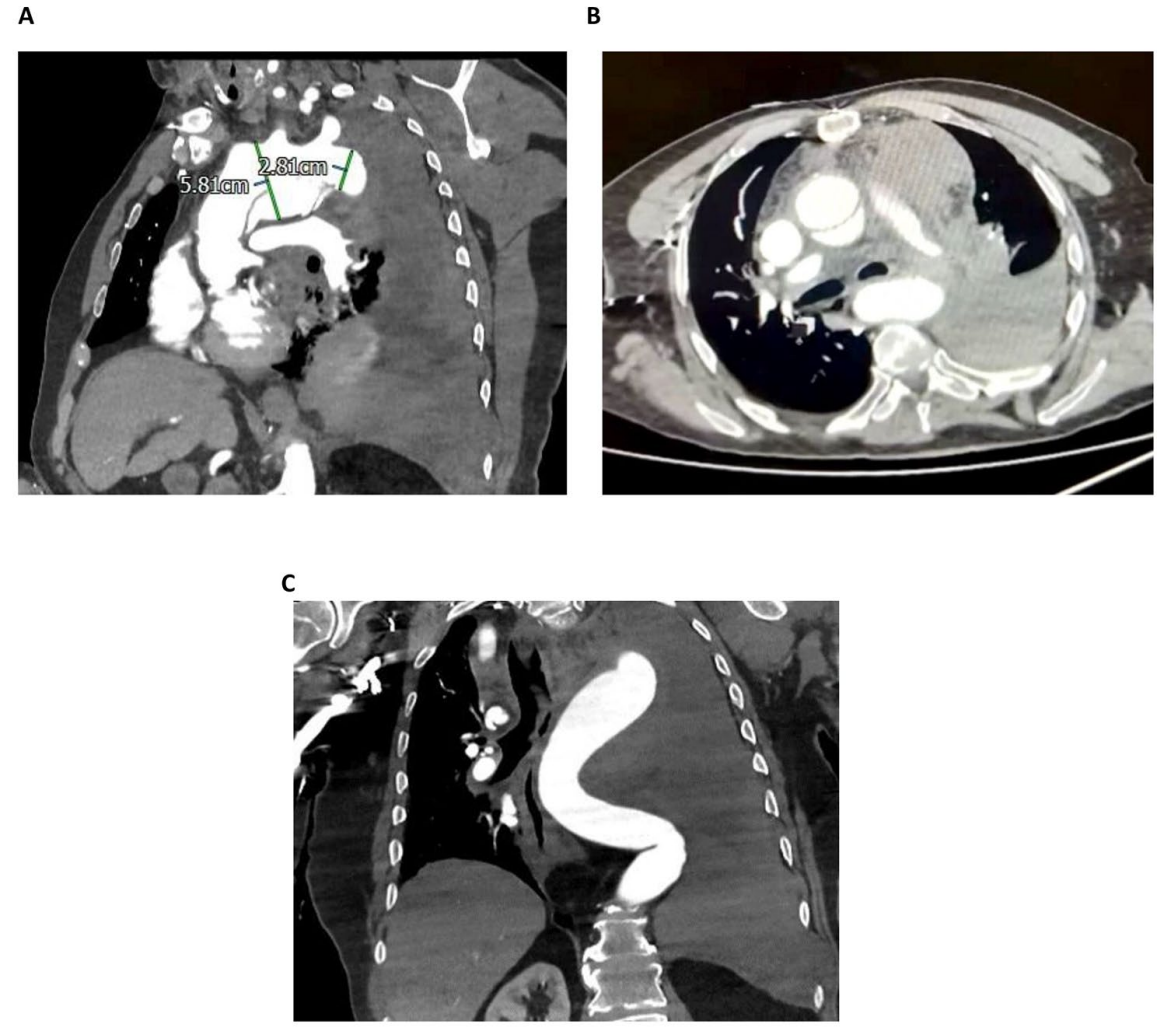
(**A**) CECT sagittal image: Aortic dissecting aneurysm extending from ascending aorta (distal to previous composite graft) to proximal descending aorta, intimal tear in the aortic arch, mid aortic arch measured 5.81 cm in diameter, and proximal descending aorta (landing zone for stented portion of Thoraflex) measured 2.81 cm in diameter; (**B**) CECT axial image: Aortic dissection of ascending aorta with the presence of true and false lumen; (**C**) CECT coronal image: Large left haemothorax and tortuous descending aorta

A 72-year-old gentleman with previous surgery of aortic root replacement 9 years ago (23 mm Carbomedics composite valve graft) for aortic root aneurysm with severe aortic regurgitation, presented at emergency department with sudden onset of tearing chest pain. He has medical history of hypertension and ischemic stroke without residual neurological deficit. Clinical examination showed blood pressure of 110/70 mmHg, heart rate of 90 bpm, respiratory rate of 26 breaths per minute, and absent air entry of left lung. Otherwise, cardiovascular and neurological examinations were unremarkable. Electrocardiogram did not show features of acute myocardial infarction. Contrast enhanced computed tomography (CECT) scan revealed acute aortic dissecting aneurysm extending from ascending aorta (distal to previous composite graft) to proximal descending aorta, intimal tear in the aortic arch (but unable to determine the location of leak), a large left haemothorax, and tortuous descending aorta; the diameter of mid aortic arch and proximal descending aorta (landing zone) were measured (Fig. 1A, B, C).

Patient was then arranged for emergency redo aortic surgery with FET technique. Transoesophageal echocardiography (TOE) showed good biventricular function, no pericardial effusion, and no valvular abnormalities. However, patient developed hypotension with blood pressure of 60/40 mmHg, and hence cardiopulmonary bypass (CPB) was established instantly via right femoro-femoral cannulation. The core temperature was cooled to 20 °C.

Redo-sternotomy and dissection of mediastinal tissue was performed to expose and identify mediastinal structures. In this case, we encountered difficulty in identifying the left subclavian artery (LSA) due to its posterior displacement caused by dissecting aneurysm of aortic arch and extensive surrounding hematoma. Hence, we decided to try locate it again later during resection of aortic arch in the state of circulatory arrest.

**Fig. 2:**
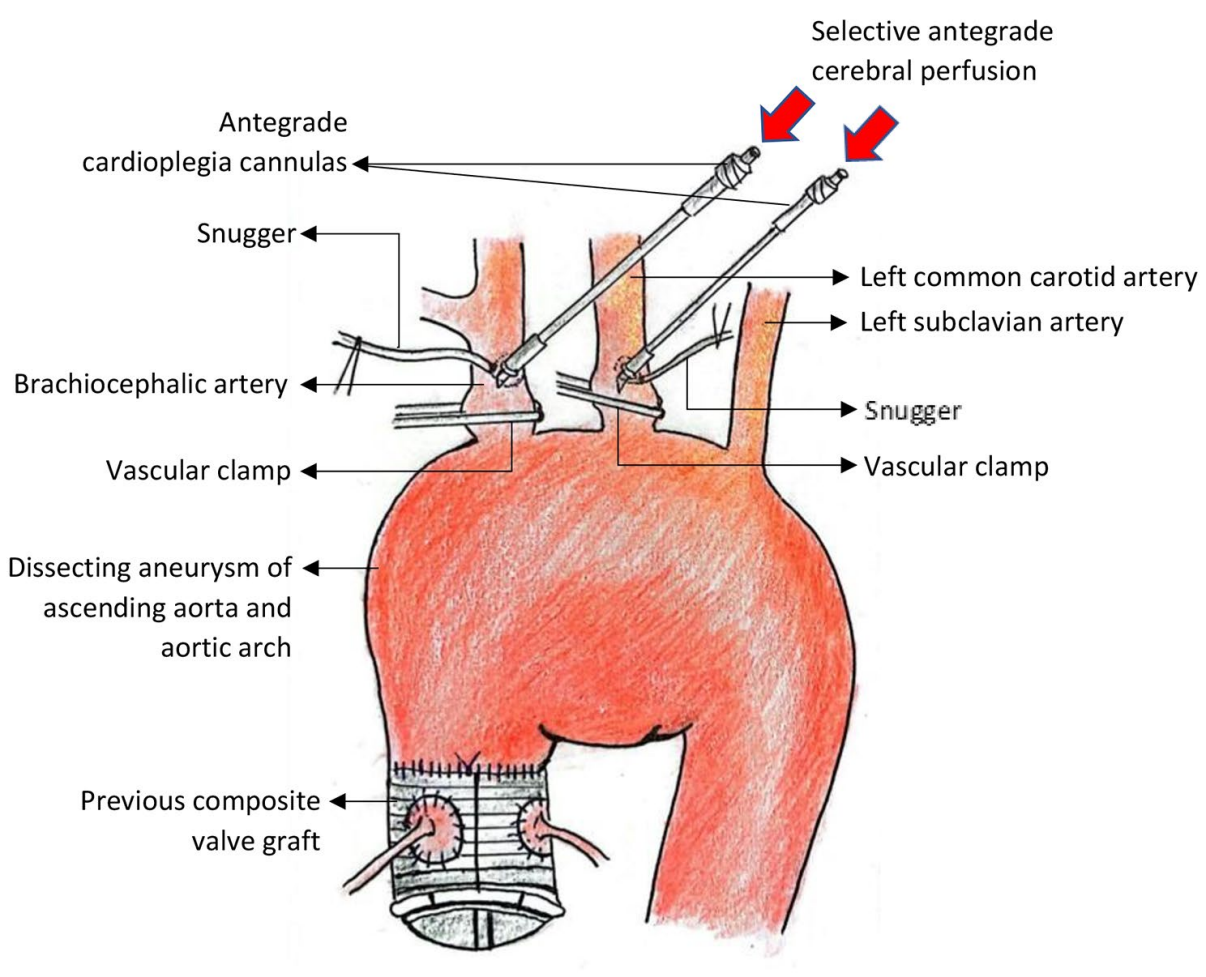
Brachiocephalic and left common carotid arteries were cannulated with antegrade cardioplegia cannulas and were clamped followed by initiation of SACP before aorta is opened. LSA was unidentified at this stage and hence it was not clamped during SACP

Right superior pulmonary vein vent was inserted. It was followed by cannulation of brachiocephalic and left common carotid arteries (at the sites without atherosclerotic plaque or dissection) using antegrade cardioplegia cannulas for delivery of selective antegrade cerebral perfusion (SACP). The trick to avoid posterior vessel wall injury during cannulating the head and neck vessels was to make a small incision at brachiocephalic and left common carotid arteries by using scalpel blade no.11, followed by insertion of cardioplegia cannulas for SACP delivery. Once the core temperature has reached 20 °C, hypothermic circulatory arrest (HCA) was initiated. The brachiocephalic and left common carotid arteries were clamped and SACP of the brain with 10 ml/kg/min of cold blood was commenced [5] (Fig. 2).

**Fig. 3:**
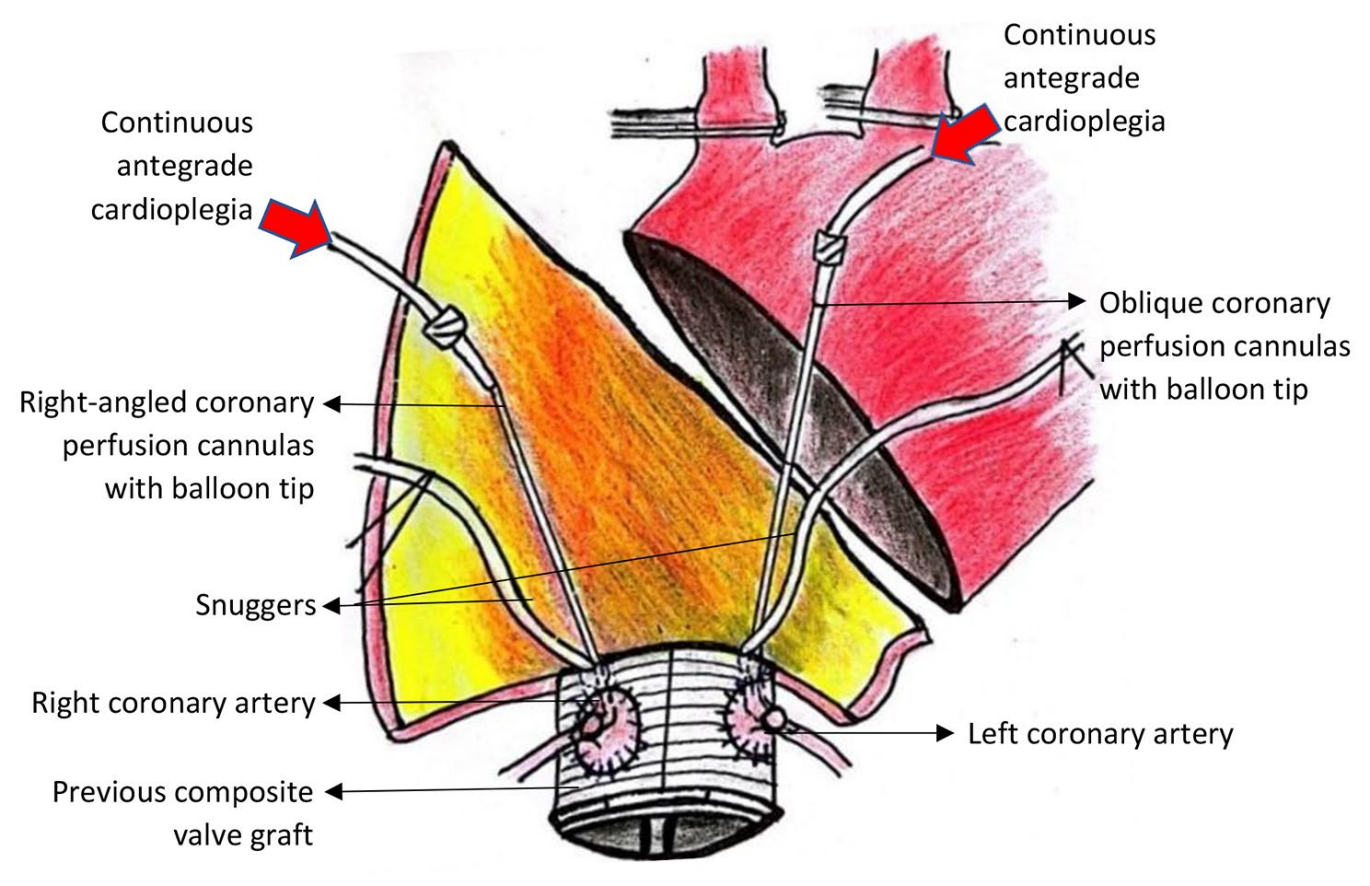
Right-angled coronary perfusion cannula with balloon tip was placed at right coronary ostia whereas oblique cannula was inserted at left coronary ostia. Continuous antegrade cold blood cardioplegia was administered

The ascending aorta was transected and incised proximally towards previous composite valve graft. Coronary perfusion cannulas with balloon tip were placed at coronary ostia and continuous antegrade cold blood cardioplegia was administered (Fig. 3). Then, the aorta was incised distally towards the distal aortic arch to complete resection of intimal tear and debranch the brachiocephalic as well as left common carotid arteries. We identified the LSA but we were unable to dissect LSA from surrounding tissue and retract it for anastomosis due to posterior displacement and extensive hematoma. The carbon dioxide insufflation was administered in the operative field to minimize the risk of air embolism.

As the measurement of proximal descending aorta on CECT thorax was 2.8 cm in diameter, Thoraflex Hybrid Plexus 4 stent graft 28 mm x 100 mm was deployed. The distal anastomosis of the sewing collar of stent graft to the aortic stump (Zone 2) was performed using single-layered 3 − 0 continuous Prolene sutures. Extracorporeal circulation was then resumed. The Thoraflex Hybrid Plexus graft was de-aired and clamped. Subsequently, the arterial line was transferred to the graft via its perfusion side arm in order to switch peripheral to central arterial cannulation. At this stage, there was no significant leakage from the distal anastomotic site.

Brachiocephalic artery and left common artery were anastomosed to the branches of the aortic arch stent graft with continuous 5 − 0 Prolene sutures. Vascular clamps were released after deairing the arch vessels and rewarming initiated.

The proximal end of Thoraflex Hybrid Plexus graft was trimmed to appropriate length and anastomosed to previous composite graft with continuous 4 − 0 Prolene sutures. Once the heart has deaired, aortic cross-clamp was released. Left pleural was opened and 2 L of old blood were removed from pleural cavity. The LSA was oversewn prior to coming off CPB. After weaning from CPB, we noted that the differential systolic blood pressure between left and right radial artery was small (20–30 mmHg). Hence, we did not proceed with revascularisation of left subclavian artery. Protamine was given to reverse the effects of heparin.

**Fig. 4:**
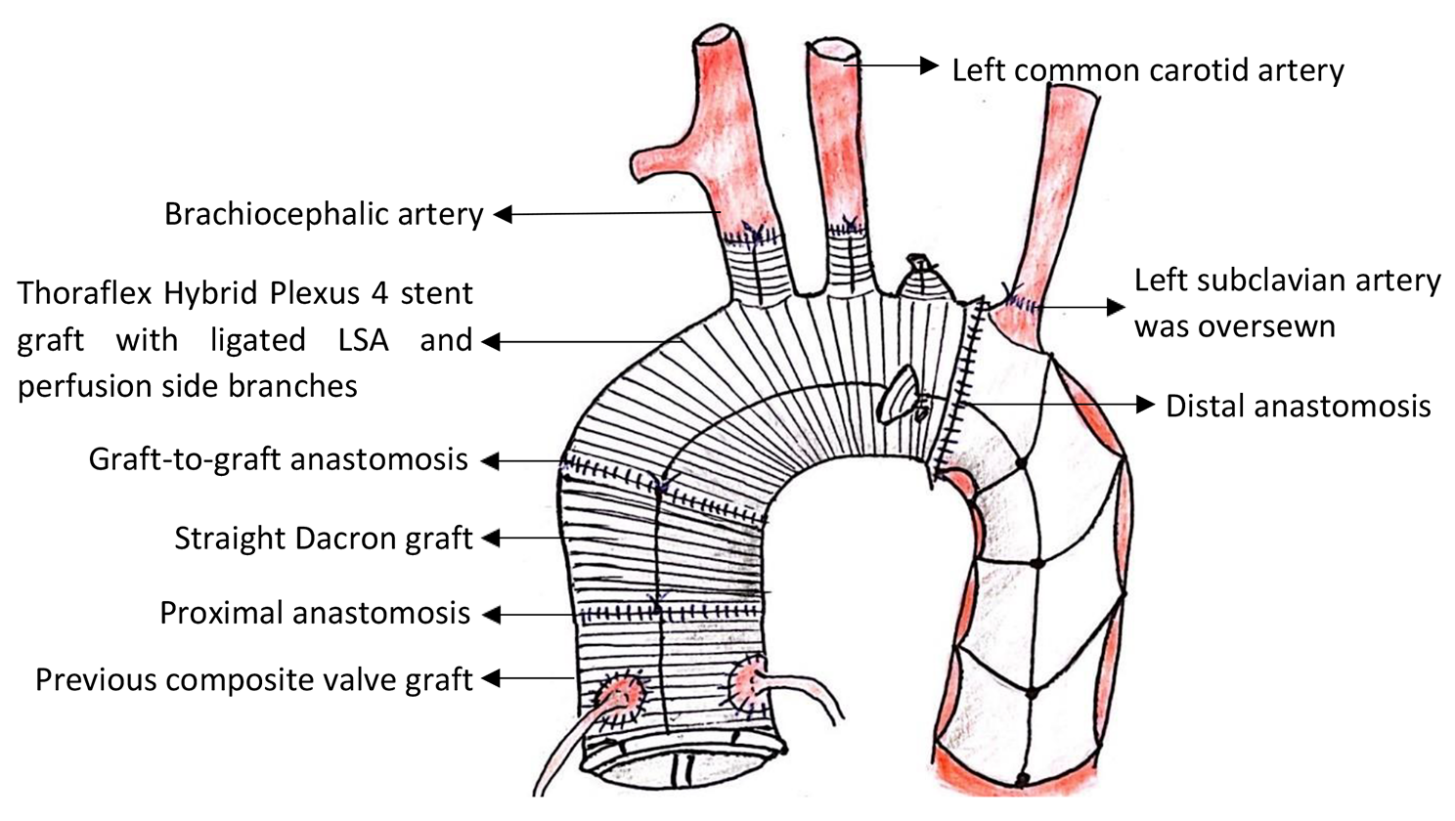
Illustration of FET procedure in this case with 2 separate grafts (straight Dacron graft and Thoraflex Hybrid Plexus 4 stent graft)

However, there was profuse bleeding from posterior portion of proximal and distal anastomoses. We were unable to control the bleeding with additional stitches. Cardiopulmonary bypass was re-established, the core temperature was cooled to 34 °C and antegrade cardioplegia was administered after application of aortic cross-clamp. Proximal aortic anastomosis was detached to expose the bleeding points at distal anastomosis. Teflon strips with interrupted 3 − 0 Prolene mattress sutures were used to reinforce the suture line of the posterior portion of distal anastomosis and the bleeding was significantly reduced. A new straight Dacron graft 30 mm was utilised for proximal anastomosis with previous composite graft by using Prolene 4 − 0. Antegrade cardioplegia was administered through the straight graft and there was no significant leakage from proximal anastomotic site. This was followed by graft-to-graft anastomosis of straight Dacron graft to Thoraflex stent graft with Prolene 4 − 0 (Fig. 4).

After the heart was deaired, aortic cross-clamp was released and slowly weaned from cardiopulmonary bypass. Temporary epicardial pacing wires were anchored on the cardiac surface. Protamine and blood products were given. Haemostasis was achieved with application of haemostatic agents (Surgicel and Floseal) and gauze packing. Post-operative TOE showed good biventricular function and no valvular abnormality.

**Fig. 5:**
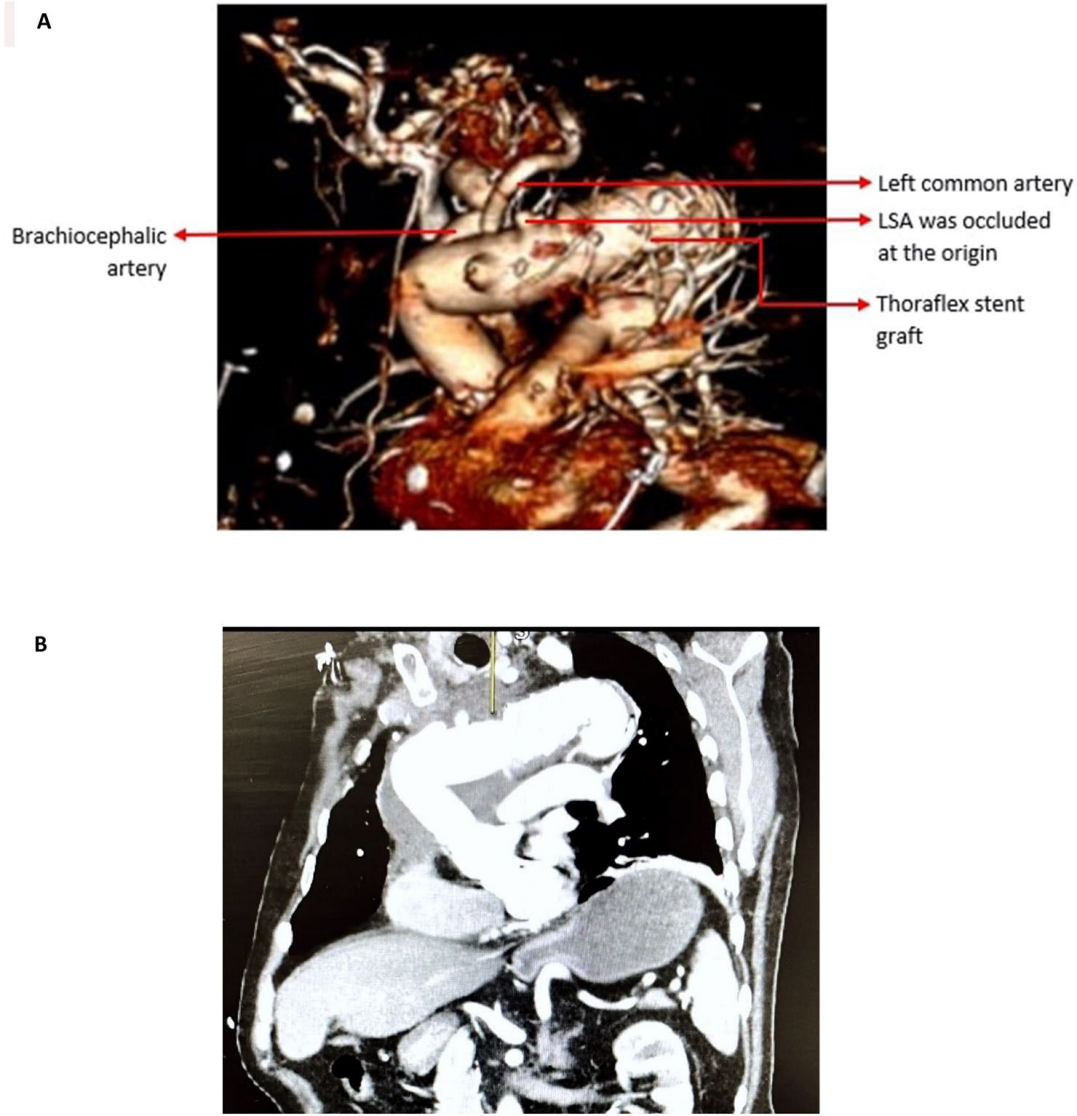
(**A**) CECT 3-dimensional image: Replacement of ascending aorta and aortic arch with reimplantation of brachiocephalic as well as left common arteries by using straight Dacron graft and Thoraflex stent graft. LSA was occluded at the origin. (**B**) CECT oblique image: Satisfactory repair of ascending aorta and aortic arch without residual dissection or aneurysm

**Fig. 6:**
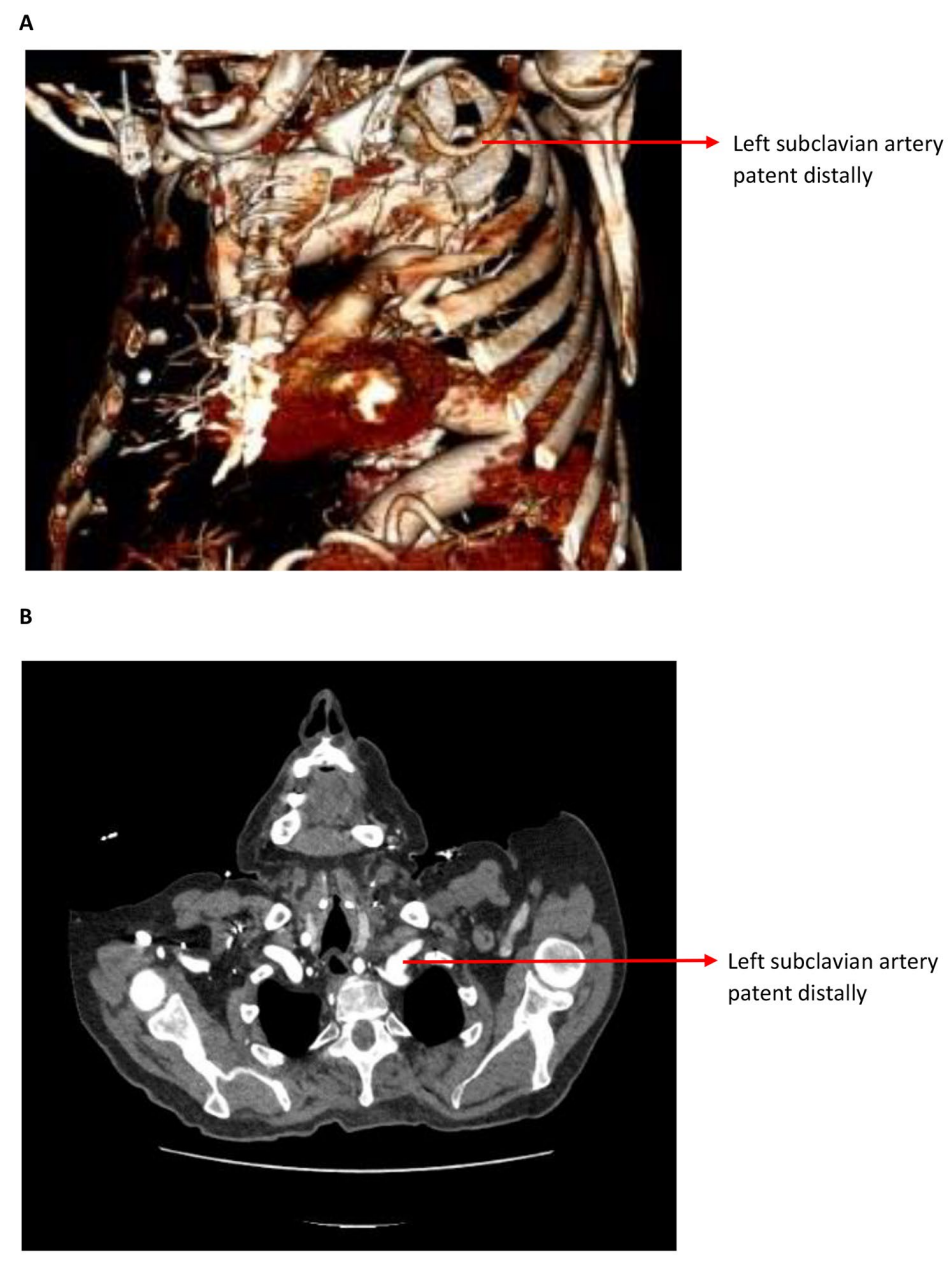
(**A**) CECT 3-dimensional image: LSA patent distally; (**B**) CECT transverse image: LSA patent distally

Clinical assessment after surgery showed his left radial pulse was palpable with normal capillary refill time. His postoperative course was uneventful, no stroke and no paraplegia. The CECT scan after 1 month of FET procedure revealed satisfactory repair of ascending aorta and aortic arch with reimplantation of brachiocephalic as well as left common arteries, and the LSA was occluded at the origin (Fig. 5A, B) but LSA patent distally (Fig. 6A, B). His postoperative follow-up at outpatient clinic was uneventful.

## Discussion

This is a case of acute Type A aortic dissecting aneurysm of ascending and aortic arch complicated with left haemothorax. Thus, this patient is indicated for emergency open surgical repair [6, 7] of ascending aorta and aortic arch. The CECT scan showed intimal tear at aortic arch, but it was unable to determine the location of the leak. Hence, TEVAR and conventional total arch replacement would not be feasible because these interventions may not adequately deal with the leak. Owing to these reasons, we have decided to proceed with redo aortic surgery with FET technique which allows full coverage of aortic arch and proximal descending aorta.

In terms of cannulation, axillary is preferred to femoral cannulation whenever possible to avoid stroke or retrograde malperfusion [8, 9]. Nevertheless, this patient developed hemodynamic instability, and hence we opted for peripheral femoro-femoral cannulation for rapid access to establish CPB instantly rather than axillary or central cannulation [10–12, 25]. Apart from that, femoro-femoral CPB allows us to decompress the heart before resternotomy to avoid re-entry injury [13]. 

In regard to cerebral protection during HCA, we cannulated the brachiocephalic and left common carotid arteries with cardioplegia cannula at the sites where preoperative CECT scan showed neither dissection nor atherosclerotic plaque, followed by clamping the origin of these vessels and commencing bilateral SACP before the aorta is opened. This approach not only aims to provide cerebral blood flow to both hemispheres but also to minimise the risk of air embolism to the brain leading to ischemic stroke or post-operative cognitive dysfunction. On the other hand, in patients who preoperative CECT scans showed dissection or atherosclerotic plaque of head and neck blood vessels, we would open the aorta during HCA and cannulate the true lumen of transected arch vessels under direct vision for SACP. This is to avoid cerebral embolism or malperfusion [14]. Other surgeons proposed not to aspirate during cannulation of arch vessels to avoid air embolism to brain [15]. Other methods to reduce the incidence of air embolism including flooding the surgical field with carbon dioxide [16, 17] and adequate deairing [18]. At our centre, we practice bilateral SACP for majority of the procedures involving aortic arch. Studies showed that bilateral SACP was associated with improved overall survival and lower cerebrovascular events in patients with SACP durations over 40–50 min as compared to unilateral SACP [19–22]. However, some studies revealed that unilateral SACP was non-inferior to bilateral SACP [23, 24]. Nevertheless, it is still a matter of debate whether bilateral SACP should preferentially be applied or whether the use of unilateral SACP is sufficient. Hence, the approach of SACP is depending on surgeon’s preference. In Europe, the commonest applied strategy is moderate HCA (20.1–24$^\circ$C) with the application of bilateral SACP [25]. 

Apart from cerebral protection, myocardial protection during cardiopulmonary bypass is crucial for successful recovery and improved outcomes. The primary method of myocardial protection is the combination of hypothermia and the administration of cardioplegia. There are different components and modes of cardioplegia delivery [26]. Our centre utilised continuous cold blood antegrade cardioplegia in all aortic cases. Studies concluded that continuous cardioplegia provided better myocardial protection than intermittent cardioplegia [27–29]. In this case, in spite of the cumulative aortic cross-clamp time was approximately 3 h, the heart returned to sinus rhythm spontaneously as well as weaned off cardiopulmonary bypass without inotropic support and postoperative TOE showed good biventricular function.

At our centre, we attempted to perform anatomic reconstruction of LSA for every FET procedure either by end-to-side or end-to-end anastomosis of LSA with aortic arch graft. However, in this case, we encountered difficulty to expose and manipulate LSA for anastomosis to aortic graft. Hence, the LSA was oversewn to avoid retrograde perfusion of the aortic arch by collaterals feeding the LSA in order to avoid continuous bleeding through the aortic perforation that maybe present beyond the anastomosis of Thoraflex stent graft. After weaning from CPB, we noted small differential systolic blood pressure between left and right radial artery between 20 and 30 mmHg. Thus, we did not proceed with revascularisation. The postoperative clinical assessment showed that his left upper limb was well perfused and no sign of paraplegia. According to 2010 Society of Vascular Surgery practice guideline, it suggested that patients with acute thoracic emergencies where thoracic endovascular aortic repair (TEVAR) is required more urgently and coverage of the LSA will be necessary, revascularization should be individualized and addressed according to the patient’s anatomy, urgency of the procedure, and availability of surgical expertise for LSA revascularization [30]. We apply this guideline to the LSA in total arch replacement with FET surgery for acute aortic dissection. Besides, a recent Cochrane review by Hajibandeh S. et al. demonstrated that revascularisation of the LSA was associated with a similar risk of stroke, spinal cord ischaemia, and mortality compared with no LSA revascularisation [31]. Other than oversewing or direct ligation of the left SCA, there are multiple strategies to manage difficult LSA during emergency aortic arch repair [32] including extra-anatomical bypass of aortic graft to left axillary artery through left subclavicular incision [33], or hybrid endovascular technique with deployment of the single-branched stent-graft inside left SCA under transesophageal echocardiography guidance [34]. 

In FET procedure, we usually do not reinforce the distal aortic stump with Teflon felt when anastomose to sewing collar of FET graft. This is because the stent graft covers proximal descending thoracic aorta. Therefore, it reduces the arterial pressure over distal anastomosis suture line and the risk of bleeding is low. Furthermore, there are several drawbacks of using Teflon felt in aortic surgery such as obscuring bleeding spot and making it necessary to be removed for proper haemostasis [35], formation of dense adhesions due to its proinflammatory properties that cause difficult dissection and bleeding during further interventions [36], and it may narrow the native aorta causing haemolysis and relative stenosis if multiple layers of Teflon felt are used [37]. Nevertheless, in this case, we encountered difficulty to achieve haemostasis as a result of tissue fragility. Even though we have confirmed that there was no significant bleeding from distal anastomotic site after resuming extracorporeal circulation, the aortic wall started to tear once weaning off cardiopulmonary bypass and led to profuse bleeding which was not stopped by additional stiches. Eventually, the haemostasis was achieved after application of Teflon felt. Thus, the usage of Teflon felt should be determined on a case-by-case basis and this case highlighted its benefit when used in extremely fragile aortic tissue. The other important technique to avoid profuse bleeding in proximal anastomosis of FET procedure is to use 2 separate grafts for replacement of ascending aorta plus aortic arch [38]. This method allows full exposure and access to anastomotic site between proximal aortic stump and straight Dacron graft. Hence, by administering antegrade cardioplegia, we could check any bleeding point and control haemorrhage easily especially bleeding over posterior portion of proximal anastomosis. Apart from that, by using 2 separate grafts method, it optimizes the lengths and orientation of the grafts to avoid dehiscence caused by tension, kinking, or obstruction [38]. 

## Conclusion

Emergency redo ascending and aortic arch surgery is technically challenging with high risk of morbidity and mortality. FET technique is an effective and safe treatment option in this complicated case. In addition, deliberate preoperative surgical planning and formulation of bail-out strategies to overcome any potential intraoperative difficulties or complications are crucial to reduce postoperative morbidity and mortality risks.

## Data Availability

Not applicable.

## Used

FET: Frozen elephant trunk
CECT: Contrast enhanced computed tomography scan
TOE: Transoesophageal echocardiography
CPB: Cardiopulmonary bypass
HCA: Hypothermic circulatory arrest
SACP: Selective antegrade cerebral perfusion
LSA: Left subclavian artery
TEVAR: Thoracic endovascular aortic repair
